# Comparative genomics reveals the genomic basis of race T2 emergence and heavy metal resistance in *Xanthomonas euvesicatoria* pv. *perforans*

**DOI:** 10.3389/fmicb.2025.1718089

**Published:** 2026-01-20

**Authors:** Chien-Jui Huang, Ting-Li Wu, Yu-Han Lin, Yao-Cheng Lin

**Affiliations:** 1 Department of Plant Medicine, National Chiayi University, Chiayi, Taiwan; 2 Biotechnology Center in Southern Taiwan, Academia Sinica, Tainan, Taiwan; 3 Agricultural Biotechnology Research Center, Academia Sinica, Taipei, Taiwan

**Keywords:** comparative genomics, effector, lysogenic conversion, microbial diversity, microbial evolution, plasmid

## Abstract

Bacterial spot poses a significant threat to global pepper and tomato production. Recent phylogenomic analysis of whole genome sequences has revealed that solanaceous bacterial spot-causing xanthomonads belong to five distinct phylogenetic lineages within three species, including two pathovars within *Xanthomonas euvesicatoria*, *X. hortorum* pv. *gardneri*, and *X. vesicatoria*. *X. euvesicatoria* pv. *perforans* (*Xep*) strains are highly diverse and have become predominant in many tomato production regions. In this study, recently emerged *Xep* strains from Taiwan were assigned to tomato race T2 based on differential cultivar phenotyping, with effector genotyping used as supporting predictors. To clarify the genomic features of these *Xep* T2 strains, high-quality genome sequences of two representative isolates were generated and performed comparative genomic analyses were conducted. The T2 phenotype of these strains were supported by the absence and presence patterns of race-associated effector genes in the genome assemblies. Comparative analysis against published *Xep* genomes revealed plasmid diversity, the evolution of copper resistance, and signatures of horizontal gene transfer in these *Xep* T2 strains. Notably, a region containing a complete set of copper and heavy metal resistance genes was integrated into the chromosome, providing evidence on evolution of copper resistance in *Xep* strains in Taiwan. Accordingly, these findings suggest that horizontal gene transfer, including lysogenic conversion, and genetic recombination contribute to the ongoing diversification of *X. euvesicatoria* pv. *perforans* and may facilitate adaptation and persistence in tomato production agroecosystems.

## Introduction

The genus *Xanthomonas* encompasses a diverse group of Gram-negative plant-pathogenic bacteria that collectively infect more than 400 plant species worldwide (Timilsina et al., 2020). Among these pathogens, bacterial spot disease on tomato (*Solanum lycopersicum*) and pepper (*Capsicum* spp.) is one of the most economically important and widely distributed diseases, causing significant yield losses and reducing fruit quality in major production regions (Osdaghi et al., 2021). The complexity of bacterial spot disease is reflected not only in its broad host range but also in the genetic diversity of the causal xanthomonads. Initially, bacterial spot xanthomonads were classified into two clonal groups and divided into four phenotypic races based on pathogenicity assays (Jones et al., 2004). More recently, whole-genome sequencing has revised this view by resolving the bacterial spot pathogens into multiple phylogenetic lineages across three species (Jones et al., 2004; Constantin et al., 2016; Osdaghi et al., 2021; Huang et al., 2024). These include *X. euvesicatoria* pv. *euvesicatoria* (Xee), *X. euvesicatoria* pv. *perforans* (Xep), the Taiwan lineage within *X. euvesicatoria* (*Xet*; formerly classified as atypical *Xep*), *X. hortorum* pv. *gardneri*, and *X. vesicatoria* (Jones et al., 2004; Constantin et al., 2016; Osdaghi et al., 2021; Huang et al., 2024). This phylogenomic framework has provided a foundation for reassessing epidemiology, effector repertoires, and evolutionary dynamics in bacterial spot pathogens.

Within these lineages, *Xep* has emerged as particularly diverse. Population genomic studies indicate that *Xep* diversity is shaped by recombination, horizontal gene transfer, and selection imposed by host resistance and agricultural practices (Abrahamian et al., 2021). For example, *Xep* strains from Florida that were once classified as a single group in the 1990s (Schwartz et al., 2015; Timilsina et al., 2019b) were later shown, with broader sampling and genome-scale data, to comprise six distinct phylogenetic lineages (Newberry et al., 2019; Abrahamian et al., 2021). Likewise, Australian *Xep* strains form unique phylogenetic clusters distinct from those in North America, Europe, or Asia (Roach et al., 2019). This recurrent diversification highlights the genetic plasticity of *Xep*, driven by recombination, horizontal gene transfer, and selection imposed by host resistance and agricultural practices. Recently, a recent global population-genomics study reports that *Xep* is widely distributed in hot, humid production regions and exhibits substantial genomic diversity, reflecting intercontinental dissemination followed by local expansion and regional diversification, which can produce geographic stratification (Kaur et al., 2025).

In Taiwan, bacterial spot is a longstanding and widespread disease affecting both tomato and pepper. The disease was first reported in the 1980s, and it remains one of the most significant constraints on tomato production (Leu et al., 2010; Burlakoti et al., 2018). Earlier surveys indicated that bacterial spot on tomato was predominantly caused by *Xep*, while *Xee* was more common on pepper (Leu et al., 2010; Burlakoti et al., 2018; Chen et al., 2024). However, since the late 1990s, a distinct lineage, that was denoted as *Xet* (Huang et al., 2024), was discovered in southern Taiwan and has since persisted in both tomato and pepper production areas (Huang et al., 2024; Parajuli et al., 2024). A remarkable shift in the composition of tomato-infecting xanthomonads has occurred in Taiwan over the past three decades. Burlakoti et al. (2018) reported that between 1989 and 1999, 95% of tomato isolates were *Xee*, with no *Xep* detected. By contrast, *Xep* was first reported on tomato in Taiwan in 2010 (Leu et al., 2010), and retrospective analyses revealed that already, 22% of isolates collected between 2000 and 2009 were *Xep* (Burlakoti et al., 2018). Since 2010, *Xep* has rapidly replaced *Xee* as the predominant tomato pathogen in Taiwan, with >99% of isolates now identified as *Xep* (Burlakoti et al., 2018; Chen et al., 2024). These findings illustrate a clear pathogen replacement event, likely driven by adaptation of *Xep* lineages to tomato hosts and selective pressures from disease management strategies.

In addition to species-level shifts, transitions at the level of tomato races have also been observed (Timilsina et al., 2016; Burlakoti et al., 2018). Tomato races are traditionally defined phenotypically by differential host responses on a panel of tomato cultivars, reflecting effector-resistance gene interactions. Among *Xep*, two races have been well characterized: race T3 and race T4. Race T3 isolates typically elicit hypersensitive responses (HR) responses on tomato cultivars such as Hawaii-7981 and LA716, consistent with recognition mediated by *Xv3* and *Xv4*, and commonly associated with intact *xopAF* (*avrXv3*) and *xopJ4* (*avrXv4*) (Astua-Monge et al., 2000a; Astua-Monge et al., 2000b). Race T4 isolates are defined by the corresponding HR phenotype and are often predicted by retention of *xopJ4* together with a disrupted *xopAF* allele (Astua-Monge et al., 2000a; Astua-Monge et al., 2000b). Race T3 was the first to emerge in tomato-infecting *Xep*, but it was soon replaced by race T4, which has since become dominant in many regions, including Taiwan (Timilsina et al., 2016; Burlakoti et al., 2018; Klein-Gordon et al., 2021; Chen et al., 2024). This pattern exemplifies how the gain, loss, or modification of key effectors can drive shifts in race composition under resistance gene deployment.

Despite these insights, whether other tomato race phenotypes could occur within *Xep* has remained unclear. Historically, races T1 and T2 were associated with *Xee* and *X. vesicatoria*, respectively (Osdaghi et al., 2021). Race T2 is defined by the phenotype that no HR is observed on the differential cultivars Hawaii-7998, Hawaii-7981, LA716, and Bonny Best (Jibrin et al., 2022). Until recently, *Xep* was primarily linked with T3 and T4. Our preliminary evidence suggests that a subset of *Xep* isolates displays a race T2 phenotype, and effector profiles (e.g., absence of *avrRxv* and *xopJ4* with a disrupted *xopAF*) provide supporting, predictive markers rather than a definition of race. In this study, the in-depth investigation of *Xep* isolates exhibiting the race T2 phenotype therefore represents a significant expansion of known race diversity within this pathovar.

Beyond race-associated effectors, mobile genetic elements such as plasmids, prophages, and genomic islands play crucial roles in shaping *Xep* genomes. Virulence genes including effectors (*xopJ2a*, *xopJ2b*) are often plasmid-borne, and plasmid recombination can lead to diverse plasmid architectures while maintaining key effectors (Minsavage et al., 1990; Thieme et al., 2005; Roach et al., 2019; Huang et al., 2021; Huang et al., 2024). Likewise, prophages have been shown to mediate lysogenic conversion, contributing to both the gain and loss of effectors such as *xopJ4* (Huang et al., 2024). Moreover, copper resistance in *Xep* and *Xet* is frequently plasmid-encoded, but recent evidence suggests chromosomal integration of resistance clusters, raising questions about stability, mobility, and fitness trade-offs (Kaur et al., 2025). A deeper understanding of how mobile elements contribute to effector turnover, race emergence, and resistance adaptation is critical for predicting future diversifying trajectories of *Xep* populations.

In this study, we report high-quality complete genome sequences of two newly emerged *Xep* strains from Taiwan, which were assigned to tomato race T2 based on phenotypic evidence. By integrating phylogenomics, effector mining, and comparative genomic analyses of plasmids, prophages, and resistance islands, we demonstrate how these strains differ from previously characterized *Xep* lineages. Specifically, our results provide new insights into plasmid diversity, the chromosomal integration of copper resistance clusters, and prophage-mediated effector dynamics. These findings contribute to a broader understanding of how recombination, horizontal gene transfer, and agricultural practices shape the evolution of *Xep* race T2 strains, with direct implications for disease management in tomato production systems.

## Materials and methods

### Collection and culture of *Xep* strains

*Xep* strains, which were collected previously (Lai et al., 2021) and in this study, were selected for genetic and phenotypic analysis based on the year and location of isolation (Table 1). All strains were isolated from tomato leaves with symptoms of bacterial spot. The *Xep* strains were cultured on nutrient agar (Difco) at 28 °C and maintained at −80 °C in Lysogeny broth (Difco) with 20% glycerol.

**Table 1:** Genotypes and phenotypes of *Xanthomonas euvesicatoria* pv. *perforans* strains.

Strain	Isolated year	Host	*AvrXv3* ^a,b^	*XopJ4* ^b^	*XopQ* ^b^	*XopJ2a* ^b^	*XopJ2b* ^b^	Tomato race	CuR^c^
XTN7	1996	Tomato	+	+	+	−	+	T3	+
XTN168	1998	Tomato	ES	+	+	−	+	T4	+
XTN169	1999	Tomato	ES	+	+	−	+	T4	+
XTN170	1999	Tomato	ES	+	+	−	+	T4	+
XTN171	1999	Tomato	ES	+	+	−	+	T4	+
XVT-278	2006	Tomato	+	+	+	−	+	T3	+
XVT-280	2008	Tomato	ES	+	+	−	+	T4	+
XVT-290	2013	Tomato	ES	+	+	−	+	T4	+
XVP-244	2016	Pepper	ES	+	+	−	+	T4	+
T0709-01	2016	Tomato	ES	+	+	−	+	T4	+
T0709-03	2016	Tomato	ES	+	+	−	+	T4	−
XpT2	2016	Tomato	ES	+	+	−	+	T4	−
XpT12	2016	Tomato	ES	+	+	−	+	T4	+
XpT13	2016	Tomato	ES	+	+	−	+	T4	+
XpT14	2016	Tomato	ES	+	+	−	+	T4	+
XpT35	2016	Tomato	ES	+	+	−	+	T4	+
2021 T5-1	2021	Tomato	ES	+	+	−	+	T4	+
2021 T5-2	2021	Tomato	ES	+	+	−	+	T4	+
A2-1	2022	Tomato	ES	−	+	−	+	T2	+
A6-2	2022	Tomato	ES	−	+	−	+	T2	+
A6-4	2022	Tomato	ES	−	+	−	+	T2	+
B2-1	2022	Tomato	ES	−	+	−	+	T2	+
C1-1	2022	Tomato	ES	−	+	−	+	T2	+
C2-1	2022	Tomato	ES	−	+	−	+	T2	+
C4-1	2022	Tomato	ES	−	+	−	+	T2	+
D-2	2022	Tomato	ES	+	+	−	+	T4	+
D-3	2022	Tomato	ES	+	+	−	+	T4	+

^a^ES, existence of an early stop codon.

^b^“+” indicates presence and “–” indicates absence of the corresponding effector gene (as determined by PCR/sequence-based screening).

^c^Cu^R^, copper resistance. +, resistant; −, sensitive.

### Genotypic and phenotypic characterization

Assignment of strains into tomato races was determined using differential cultivar assays of the hypersensitive responses (HR) as described by Chen et al. (2024). Detection of *xopAF* and *xopJ4* (Timilsina et al., 2016) was used as supporting evidence and as predictive markers because these loci often correlate with race phenotypes. Absence/presence of early stop codons in amplified *xopAF* fragments was determined by Sanger sequencing (Genomics BioSci & Tech Co., New Taipei, Taiwan). Four differential cultivars were *S. lycopersicum* Bonny Best (susceptible to all races), Hawaii-7998 (resistant to tomato race 1), Hawaii-7981 (with resistance gene *Xv3*), and *Solanum pennellii* LA716 (with resistance gene *Xv4*), and races T1, T2, T3, T4, and T5 were determined according to susceptibility and the HR on the differential tomato cultivars (Jibrin et al., 2022). In addition to classification of tomato races, presence of two *xopJ2* homologs, *xopJ2a* and *xopJ2b*, in *Xep* strains were detected by specific primers as described previously (Timilsina et al., 2016; Klein-Gordon et al., 2021).

Copper sensitivity tests were performed according to the two methods described by Lai et al. (2021) and Kaur et al. (2024). The *Xep* strains were first cultured overnight on NA medium. For copper susceptibility testing, strains were then streaked onto NA plates supplemented with 0, 0.4, 0.6, and 0.8 mM CuSO_4_. Copper resistance phenotypes were defined by the highest CuSO₄ concentration supporting visible growth (Behlau et al., 2013; Marin et al., 2019): sensitive (no growth at 0.6 mM), tolerant (growth at 0.6 mM but not 0.8 mM), and resistant (growth at 0.8 mM). Moreover, bacterial suspensions were prepared and adjusted to 1 × 10^6^, 1 × 10^7^, and 1 × 10^8^ CFU/mL and 10 μL of each dilution was spotted onto NA supplemented with 0.8 mM CuSO_4_. The inoculated plates were incubated, and bacterial growth was examined.

### Oxford Nanopore Technologies and Illumina sequencing, genome assembly and gene annotation

Genomic DNA extraction and sequencing were performed as described previously (Huang et al., 2021; Huang et al., 2024), with key materials summarized as follows: High-molecular-weight DNA was prepared and assessed using the QuantiFluor^®^ dsDNA System. Long-read sequencing was performed using Oxford Nanopore Technologies (ONT) with the Ligation Sequencing Kit (SQK-LSK109) and PromethION Flow Cells (R9.4.1). Base calling was performed with Dorado v7.2.12. Short-read sequencing was performed on an Illumina platform, libraries were prepared using the Illumina DNA Prep Kit and sequenced on an Illumina NovaSeq 6,000. For each strain, we generated ONT long reads and Illumina paired-end short reads. ONT sequencing yielded ~435–470 thousand reads per strain with mean read length 6.4–6.9 kb and read N_50_ 14.6–15.8 kb (Supplementary Table S1). Illumina sequencing yielded ~45–50 million 150 bp read pairs per genome (Supplementary Table S1). Raw read quality was assessed with FastQC v0.11.9 (Andrews, 2015). Illumina adapters/low-quality bases were trimmed with Trimmomatic v0.36 (Bolger et al., 2014); ONT reads were filtered with NanoFilt v2.6.0 (De Coster et al., 2018). ONT reads >1 kb were further corrected using the corresponding Illumina data with FMLRC v1.0.0 (Wang et al., 2018).

For each strain, long-read assemblies were produced with Canu v1.8 (Koren et al., 2017), and Flye v2.5 (Kolmogorov et al., 2019), selected for performance in repeat-rich regions. Draft assemblies were compared by whole-genome alignment (MUMer4 and BLASTN v2.10.1) (Camacho et al., 2009; Marcais et al., 2018) and resolved into a consensus assembly by selecting the highest contiguity draft and manually resolving structural conflicts via read mapping. Per-base accuracy was improved with fmlrc (v1.0.0) (Wang et al., 2018) using the cleaned Illumina reads and visually verified in IGV (Thorvaldsdottir et al., 2013). The final assemblies of each strain were manually verified for circularity. Terminal overlaps identified were confirmed by mapping Nanopore long reads across the start-end junction of the chromosome and all plasmids. Assembly completeness, duplication, and contamination were evaluated with CheckM (Parks et al., 2015) and BUSCO (v5.5.0 lineage: *xanthomonadales_odb10*) (Manni et al., 2021) (Supplementary Table S1).

Structural annotation used the locally installed NCBI Prokaryotic Genome Annotation Pipeline (PGAP) (Tatusova et al., 2016). Gene models (including start/stop codons) were inspected in Artemis (Carver et al., 2012). Predicted proteins were compared against published *Xanthomonas euvesicatoria* genomes with BLASTP (Camacho et al., 2009) to refine and validate annotations. Functional annotation combined Bakta (v1.8.2) (Schwengers et al., 2021; UniProt Consortium, 2025), and Prokka (v1.14.6) (Seemann, 2014). Approximately 20% of protein-coding genes remained annotated as hypothetical. The complete bioinformatic workflow, including specific command-line parameters and software versions, is documented in our GitHub repository^1^.

### Taxonomic identification and whole-genome similarity analysis

We assessed genome variation at two levels: (i) nucleotide-level similarity among complete genomes and (ii) protein-coding gene content. Our workflow was adapted from the approaches described in Huang et al. (2021) and Huang et al. (2024). The assembled chromosomes and plasmids were compared with 132 published *Xanthomonas* genomes (Supplementary Table S2) (da Silva et al., 2002; Thieme et al., 2005; Potnis et al., 2011; Jacques et al., 2013; Gagnevin et al., 2014; Schwartz et al., 2015; Bansal et al., 2017; Merda et al., 2017; Richard et al., 2017; Lopez et al., 2018; Timilsina et al., 2019a; Timilsina et al., 2019b; Studholme et al., 2020; Hu et al., 2021; Huang et al., 2021; Chen et al., 2024; Huang et al., 2024; Parajuli et al., 2024) to identify conserved and strain-specific regions (Barak et al., 2016; Constantin et al., 2016; Bansal et al., 2018; Fan et al., 2022; Huang et al., 2024). Pairwise whole-genome similarity was estimated with FastANI v1.20 (default parameters) (Jain et al., 2018). Species assignments and nomenclature were corroborated using the Genome-to-Genome Distance Calculator (GGDC) (Meier-Kolthoff et al., 2022). Phylogeny was assigned according to the thresholds defined by established genomic standards (Chun et al., 2018; Huang et al., 2024) (see Footnote 1).

### Phylogenomic analysis

To further resolve the evolutionary relationships of these two strains, orthologous gene families were inferred using two complementary datasets. Protein-coding genes were compared by all-against-all BLASTP (*e*-value ≤ 1*e*^−5^) (Camacho et al., 2009) and the resulting similarity graph was clustered with the Markov Cluster Algorithm (MCL; inflation = 5) (Enright et al., 2002). To maximize accuracy in tree construction, we analyzed additional 36 *Xanthomonas* strains with complete, high-quality assemblies or belonging to important phylogenetic positions (Supplementary Table S3). MCL clustering identified 1,512 single-copy core orthologous families. Each family was aligned with MUSCLE (Edgar, 2004), trimmed with trimAl (Capella-Gutierrez et al., 2009), and concatenated into a supermatrix. A maximum-likelihood phylogeny was inferred with RAxML-NG v1.0.1 (model LG + G8 + F; 1,000 bootstrap replicates; seed = 2) (Kozlov et al., 2019) and the resulting tree was visualized in FigTree^2^.

To capture a wider diversity of gene content, we extended the analysis to 145 *Xanthomonas* strains (Supplementary Table S2). These 145 strains were selected from recently published surveys of bacterial spot pathogens spanning a wide range of regions, including Taiwan, USA, France, Canada, and Belgium (Chen et al., 2024; Parajuli et al., 2024). MCL clustering of this dataset produced 8,606 gene families, summarized in a strain-by-family matrix. Following the definitions (Meric et al., 2014), we considered the pan-genome as the union of all families, the core genome as those shared across all strains, and the accessory genome as those present in only a subset. This expanded set was used to investigate specific gene clusters of interest. Syntenic relationships among genomes were further examined using i-ADHoRe 3.0 (Proost et al., 2012) (see Footnote 1).

### Screening of secreted effectors with DeepSecE

Protein coding genes from each genome were screened using DeepSecE (v0.1.1) to predict secreted effectors (Zhang et al., 2023). DeepSecE was run with default bacterial parameters on the command line, producing per-protein probabilities for effector secretion and a “non-secreted” class. Unless stated otherwise, we considered a protein a putative effector when the effector score ≥ 0.50 and the non-secreted probability < 0.50 (see Footnote 1).

### Horizontal transfer of copper resistance genes

The horizontal transfer of Cu^R^ genes between different xanthomonad strains was tested according to the method described by Huang et al. (2024). Briefly, strain A2-1, with copper resistance and rifampicin sensitivity, and the rifampicin-resistant mutant of the Cu^S^ strain XTN47Rif were used as a donor and a recipient, respectively. After incubation together on NA plates at 28 °C for 24 h, the bacterial cells were scraped, suspended, and plated at dilutions on NA supplemented with rifampicin (50 mg/L) to estimate the recipient population. To select transconjugants, bacterial suspensions were plated at dilutions on NA supplemented with rifampicin (50 mg/L) and 0.8 mM CuSO_4_. The conjugation frequency was calculated as the ratio of the number of transconjugants to the recipient population (Behlau et al., 2012).

## Results

### Phenotypic characteristics of Taiwanese *Xep* strains

**Figure 1:**
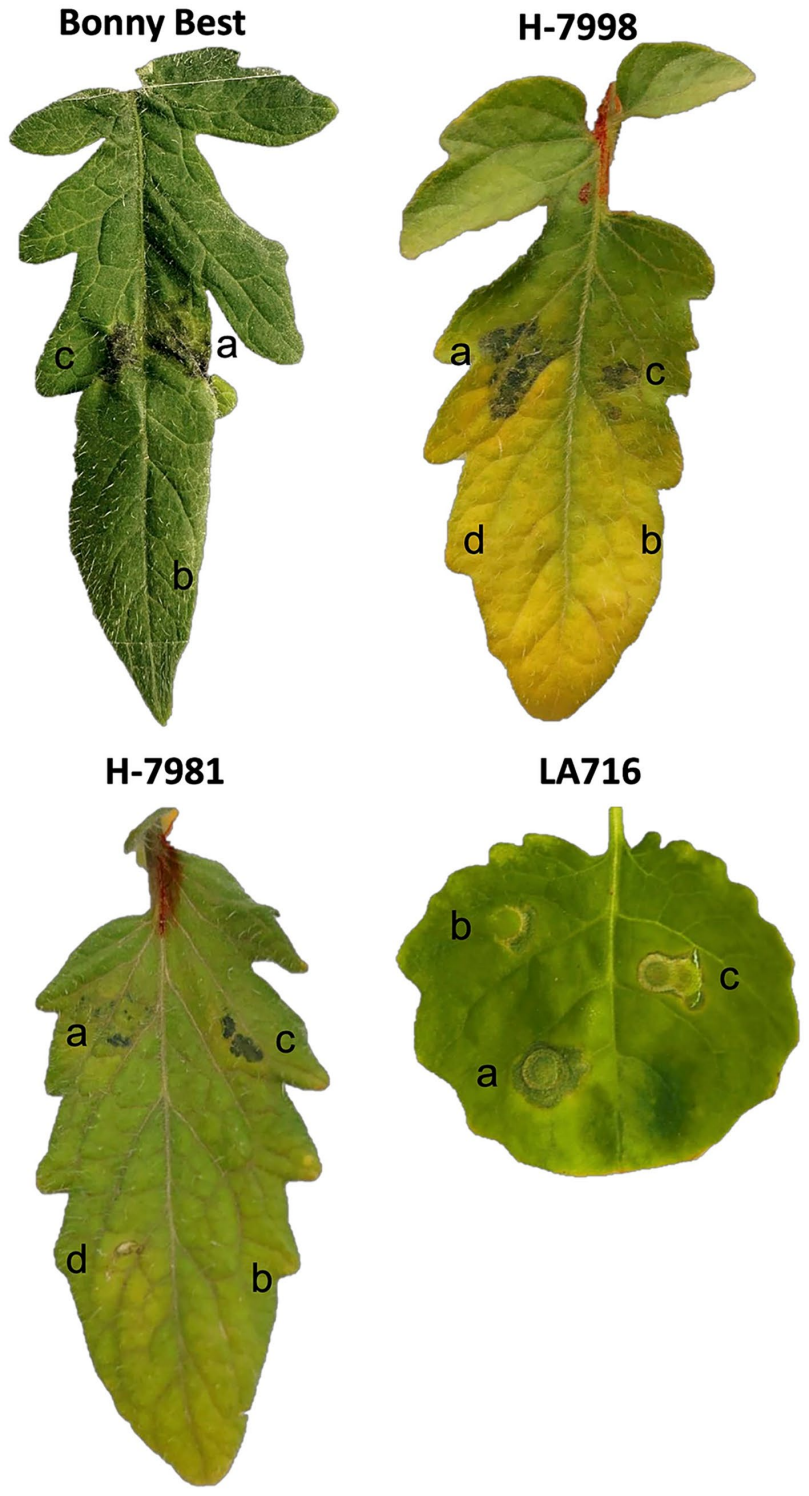
Disease symptoms and hypersensitive responses elicited on differential tomato cultivars following leaf infiltration with bacterial spot *xanthomonad* strains. Tomato cultivars are indicated above each panel. Inoculated spots and corresponding strains/effector profiles are shown: (a) Strain A2-1 (*xopJ3*^−^, *xopAF*^−^, *xopJ4*^−^, *xopJ2b*^+^), representing a newly identified race T2 isolate; (b) mock infiltration with 10 mM MgCl_2_ as a negative control; (c) strain XTN47 (*xopJ3*^−^, *xopAF*^+^, *xopJ4*^−^, *xopJ2b*^+^), representative of a race T3 strain; and (d) strain T0319-01 (*xopJ3*^−^, *xopAF*^+^, *xopJ4*^+^, *xopJ2b*^+^), representative of a race T4 strain.

We analyzed 27 *Xanthomonas euvesicatoria* pv. *perforans* (*Xep*) isolates collected in Taiwan between 1996 and 2022 (Table 1). Based on effector gene profiles, isolates were grouped into three distinct groups. Specifically, two isolates carried intact *xopAF* (*avrXv3*) and *xopJ4* (*avrXv4*) consistent with race T3. Eighteen isolates had intact *xopJ4* but harbored a premature stop codon of *xopAF*, consistent with race T4. The remaining seven isolates, collected in 2022, lacked *xopJ4* and carried a truncated *xopAF* allele, defining them as race T2. Notably, all isolates carried an intact copy of the effector gene *xopJ2b*. To confirm the phenotype of the putative race T2 strains, we infiltrated leaves of differential tomato cultivars with these isolates. None of these isolates elicited a hypersensitive response, confirming the race T2 phenotype (Figure 1). Copper resistance (Cu^R^) was prevalent among these isolates. Notably, 25 of 27 isolates grew on nutrient agar supplemented with 0.8 mM CuSO₄ (Lai et al., 2021), and all isolates collected since 2016 were Cu^R^.

### Genome sequences of *Xep* race T2 strains

We sequenced the genomes of two Taiwanese *Xep* race T2 isolates (A2-1 and C1-1) using a hybrid long-read and short-read sequencing workflow adapted from Huang et al. (2021) and Huang et al. (2024). Each assembly yielded a single circular chromosome (5.015–5.018 Mb; GC 64.9%) and a set of fully circularized plasmids (three plasmids across the two strains, 15–49 kb) (Table 2). Final contig coverage was evaluated by mapping ONT long reads and Illumina short reads independently to all assembled contigs. Each contig (chromosome and plasmids) exhibited a minimum mapping depth >200×, supporting obust coverage and reliable sequence accuracy for downstream analyses. Quality assessment with CheckM and BUSCO confirmed that both assemblies were highly complete (e.g., ≥99% completeness) and exhibited minimal contamination (≤1% contamination) (Supplementary Table S1). Comparative analysis with other recently published *Xanthomonas* genomes (Supplementary Table S2) further demonstrates that our metrics meet or exceed established quality standards for high-quality genomic resources. Gene prediction identified 4,397 genes in A2-1 and 4,313 in C1-1 from the chromosomal assemblies (including rRNA genes and pseudogenes), consistent with gene counts reported for published *Xep* genomes (Timilsina et al., 2020) (Table 2). Effector mining of the completed genomes corroborated the race T2 effector profile of these strains. Thus, the hybrid assembly strategy effectively resolved repetitive sequences and plasmid elements, yielding fully assembled genomes for both *Xep* race T2 isolates.

**Table 2:** Genome information of sequenced and reference *Xanthomonas euvesicatoria* pv. *perforans* strains.

Strain	Tomato race	Copper resistance	Size (bp)	GC %	Genes	BioProject	Isolated location	Isolated year
A2-1	T2	Cu^R^	5,017,520	64.9	4,397	PRJNA1313807 (This study)	Luzhu, TW	2022
pA2-1.1			49,091	59.8	56			
pA2-1.2			34,645	62.2	40			
pA2-1.3			30,489	61.1	33			
C1-1	T2	Cu^R^	5,015,113	64.9	4,313	PRJNA1313807 (This study)	Luzhu, TW	2022
pC1-1.2			49,091	59.8	56			
pC1-1.3			34,645	62.2	41			
pC1-1.4			15,184	64.6	17			
T0709-01	T4	Cu^R^	4,915,631	65.0	4,037	PRJNA1029321 (Huang et al., 2024)	Chiayi, TW	2016
p0709-01.1			249,567	59.6	231			
p0709-01.2			82,027	60.2	78			
p0709-01.3			28,181	63.1	36			
T0709-03	T4	Cu^S^	4,915,609	65.0	4,036	PRJNA1029321 (Huang et al., 2024)	Chiayi, TW	2016
p0709-03.1			82,042	60.2	78			
p0709-03.2			28,181	63.1	36			
XpT2	T4	Cu^S^	4,906,464	65.0	4,024	PRJNA1029321 (Huang et al., 2024)	Taipao, TW	2018
pXpT2.1			93,650	57.1	93			
pXpT2.2			82,070	60.2	78			
pXpT2.3			10,900	61.6	19			
91–118	T3	Cu^S^	4,898,349	65.0	4,350	PRJNA60021 (Potnis et al. 2015)	Florida, USA	1991

### Molecular taxonomy and phylogenomic analysis of *Xep* race T2 strains

**Figure 2:**
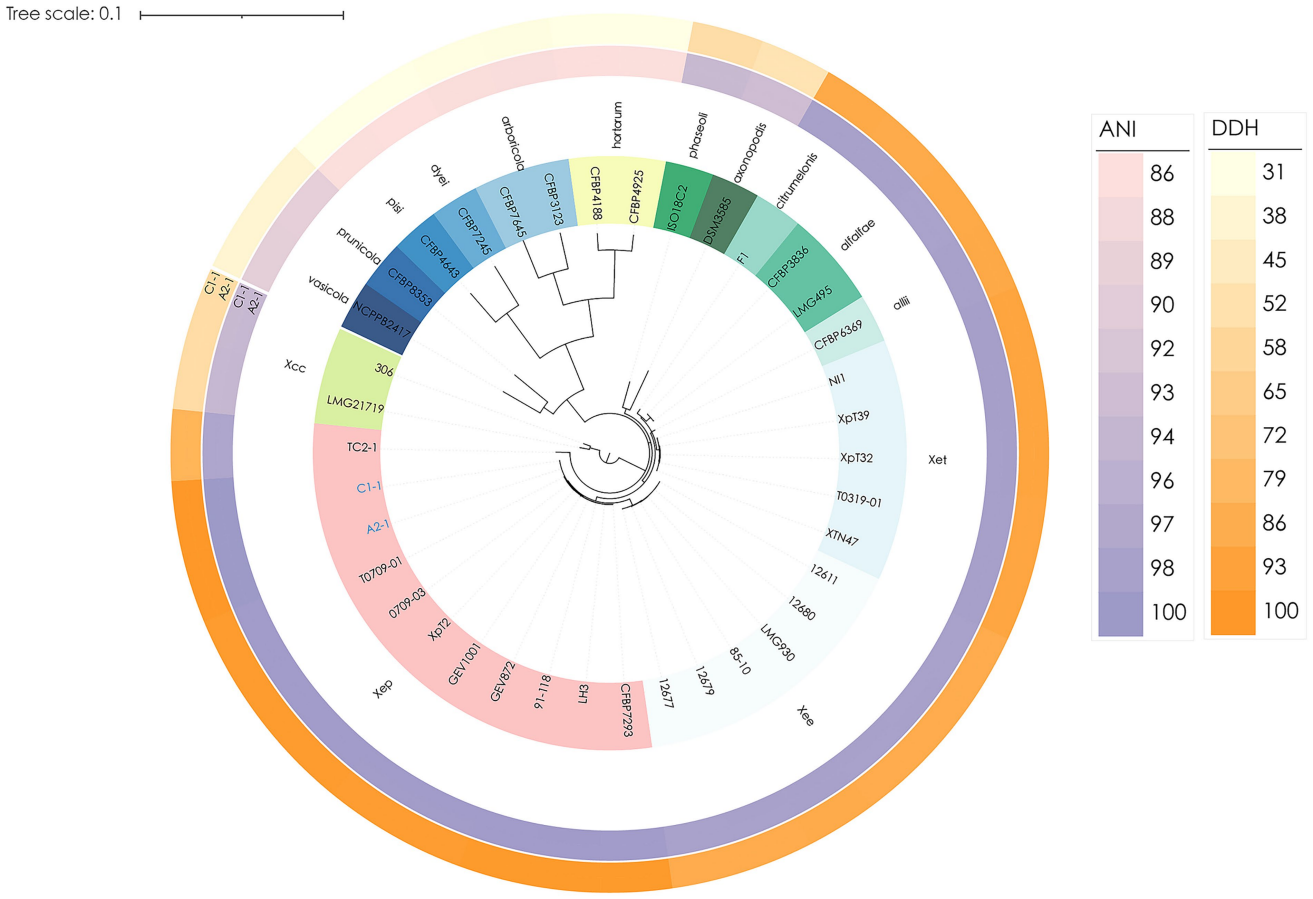
Phylogenomic placement of *Xep* race T2 strains and pairwise similarity to representative *Xanthomonas* genomes. A maximum-likelihood phylogenetic tree was inferred from a concatenated alignment of 1,512 single-copy core genes across 38 genomes, rooted with *X. citri* subsp. *citri* strain 306. The tree was constructed using RAxML-NG with 1,000 bootstrap replicates. Taiwanese race T2 strains A2-1 and C1-1 are highlighted. The outer colored rings display pairwise similarity of each genome to A2-1/C1-1: the purple ring represents average nucleotide identity (ANI), and the orange ring represents digital DNA–DNA hybridization (dDDH). Heatmap scales for ANI and dDDH values are shown on the right. Genomes are grouped and shaded by species, pathovar., or genomic lineage for reference.

To clarify the phylogenetic placement and genetic diversity, a phylogenomic tree based on 1,512 single-copy orthologous groups (38 complete genomes) placed A2-1 and C1-1 in a well-supported *Xep* clade alongside strain 91-118, distinct from the *X. euvesicatoria* pv. *euvesicatoria* (*Xee*) and the Taiwan lineage within *X. euvesicatoria* (*Xet*) (Figure 2 and Supplementary Table S3).

A complementary approach with combined comparison of the average nucleotide identity (ANI) and digital DNA–DNA hybridization (dDDH) were used to further classify the phylogeny. In bacterial genomics, species delineation typically requires ANI ≥ 95% and dDDH ≥ 70% (Chun et al., 2018). Furthermore, within *X. euvesicatoria*, strains with ANI ≥ 99.2% were grouped into the same genomic lineage following the operational threshold reported by Huang et al. (2024) and Huang et al. (2024). Strains A2-1 and C1-1 exhibited 99.7% ANI and 98.7% dDDH relative to *Xep* reference strain 91-118, confirming that these isolates belong to *X. euvesicatoria* pv. *perforans* (Figure 1). A broader screen of 145 strains with diverse geographic distribution (Chen et al., 2024; Parajuli et al., 2024) further confirm the placement of the A2-1 and C1-1 in *Xep* (Supplementary Figure S1 and Supplementary Table S3).

### Gene family distributions within the *Xep* clade

**Figure 3:**
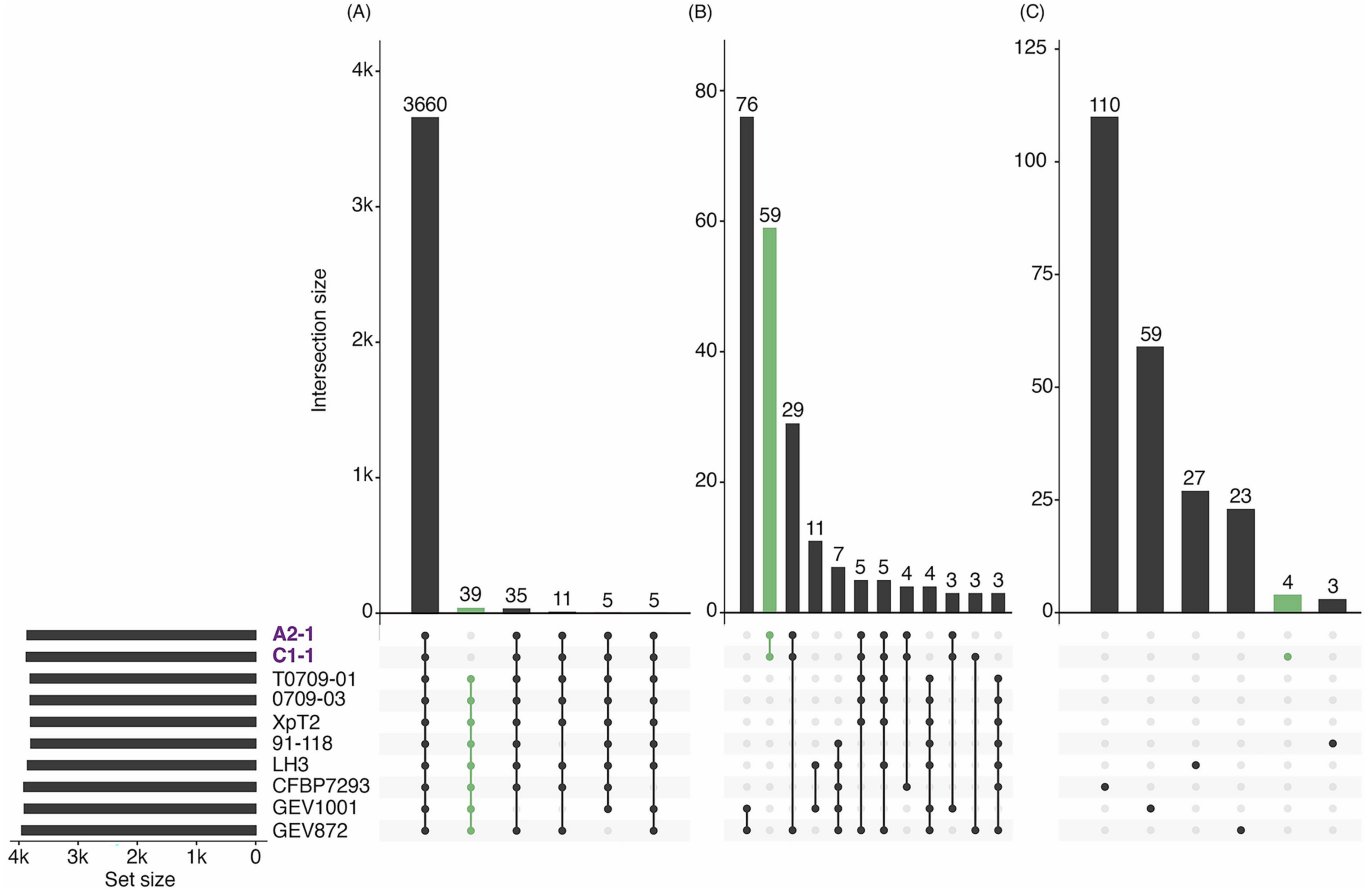
Presence–absence variation of gene families within the *Xep* clade. Orthologous gene families were clustered across 38 complete genomes (see Figure 2), and the subset corresponding to *Xep* genomes is shown here. Horizontal bars (left) indicate the total number of families present in each genome (set size). Vertical bars represent the size of intersections (intersection size) defined by the connected dots below each panel. **(A)** Core families. A total of 3,660 gene families were conserved across all *Xep* strains, representing the clade’s core genome. Smaller intersections highlight families absent from specific strains, including 39 families missing in A2-1/C1-1 and 35 families missing in GEV1001. **(B)** Subset-shared families. Families shared by only a subset of strains are shown. Among these, 59 families were exclusive to A2-1 and C1-1, while 29 were shared among A2-1, C1-1, and GEV872. **(C)** Strain-specific families. Only six of the 38 genomes carried unique families; within the *Xep* clade, C1-1 harbored four unique families, all annotated as hypothetical proteins.

To further dissect the genetic divergence, we examined gene-family distributions within the *Xep* clade containing A2-1 and C1-1 (Figures 3A–C). The core genome of this clade comprised 3,660 gene families shared by all member strains, whereas strains A2-1 and C1-1 lacked 39 gene families present in other clade members (Figure 3A). Notably, the set of families absent in A2-1 and C1-1 included the effector gene *xopJ4* (*avrXv4*) and several functions related to DNA repair/stability, environmental response, and host interaction. Initially, 59 gene families appeared to be unique to A2-1 and C1-1 within this clade (Figure 3B). However, expanding the comparison to all 38 genomes showed that only 18 of those families were truly exclusive to the two strains, while the rest occurred sporadically in strains outside the clade (Figure 3B). Among the 18 gene families identified as unique to strains A2-1 and C1-1, all those with putative functional assignments were related to virulence or host interaction. The remaining gene families were annotated as hypothetical proteins with no known function. Using the DeepSecE deep-learning pipeline for effector prediction (Zhang et al., 2023), we found that 5 of these hypothetical proteins were predicted to be candidate secreted effectors (Supplementary Tables S4, S5).

Genome-specific gene families were rare in this dataset. Among the 38 genomes analyzed, only six carried unique families, and within the focal *Xep* clade, only C1-1 contained unique genes (Figure 3C). Four families were exclusive to C1-1, all annotated as hypothetical proteins. Two of these were also present in *X. alfalfae* or *Xet*, while the other two were bona fide singletons not detected in any other genome of the 38-strain dataset. Notably, one of the C1-1 specific singletons was predicted to encode a type IV effector (Supplementary Table S5).

### Type III secretion system and effector recombination events

**Figure 4:**
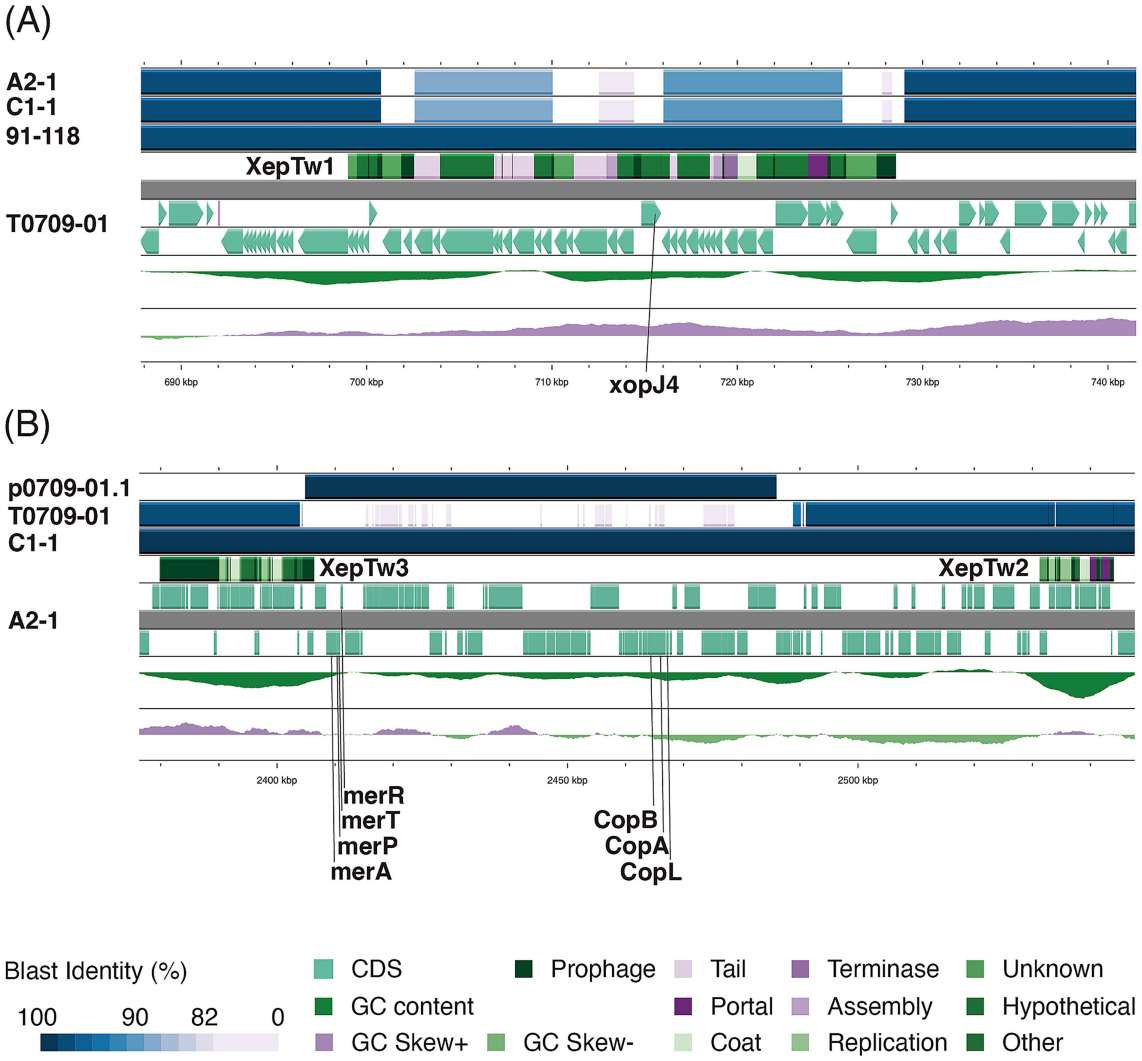
Comparative genomic context of effector and heavy metal resistance regions in *Xep* race T2 strains. **(A)** Alignment of the chromosomal region using strain T0709-01 as the reference illustrates the absence of *xopJ4* (*avrXv4*) in A2-1 and C1-1. Instead, a distinct P2-like prophage (XepTw1) is integrated at the same locus where the *xopJ4*-carrying prophage is present in other *Xep* lineages, consistent with the race T2 phenotype, based on differential assays. **(B)** Comparison of strain A2-1 with other genomes highlights the presence of two additional prophages (XepTw2 and XepTw3) flanking an ~85 kb chromosomal island encoding complete heavy metal resistance determinants, including the *copLABGF* operon and mercury resistance genes, located near the replication terminus. A third prophage XepTw4 located in within the heavy metal resistance cluster. These prophage–island associations distinguish A2-1 and C1-1 from other sequenced *Xep* strains. Color coding indicates BLASTN identity (blue gradient) and functional annotation of genomic features (green = coding sequences, dark green = prophage genes, purple = prophage structural modules, see legend).

We compared the predicted effector repertoires across clades by counting, for each genome, the fraction of predicted effectors assigned to each secretion system (Types I–VI) by DeepSecE (Supplementary Figure S2). These fractions differed among clades, indicating clade-specific effector profiles. In particular, *Xep* genomes encoded a significantly higher proportion of predicted Type III secretion system effectors (T3SEs) than *Xet*, and other *Xanthomonas*, consistent with an expanded T3SE arsenal associated with tomato adaptation. These differences underscore that each clade has evolved a distinct effector repertoire under different host and ecological pressures. Accordingly, the genome sequences of A2-1 and C1-1 were examined to characterize the allelic diversity of T3SEs that underline tomato race phenotypes in *Xep*. Genes previously implicated in host range including *xopJ3* (*avrRxv*, GenBank accession number L20423), *xopAF* (*avrXv3*, AF190120), *xopJ2a* (*avrBsT*, AF156163), *xopJ2b* (PUWL01000049.1:c1558-2622), and *xopJ4* (*avrXv4*, AF221058) were analyzed (Whalen et al., 1993; Astua-Monge et al., 2000a; Astua-Monge et al., 2000b; Timilsina et al., 2016; Jibrin et al., 2022). Both A2-1 and C1-1 lacked *avrRxv*, which is characteristic of tomato race 1 strains, and *xopJ2a*. The *xopAF* gene contained a premature stop codon, rendering it nonfunctional, and *xopJ4* was entirely absent (Figure 4A).

**Figure 5:**
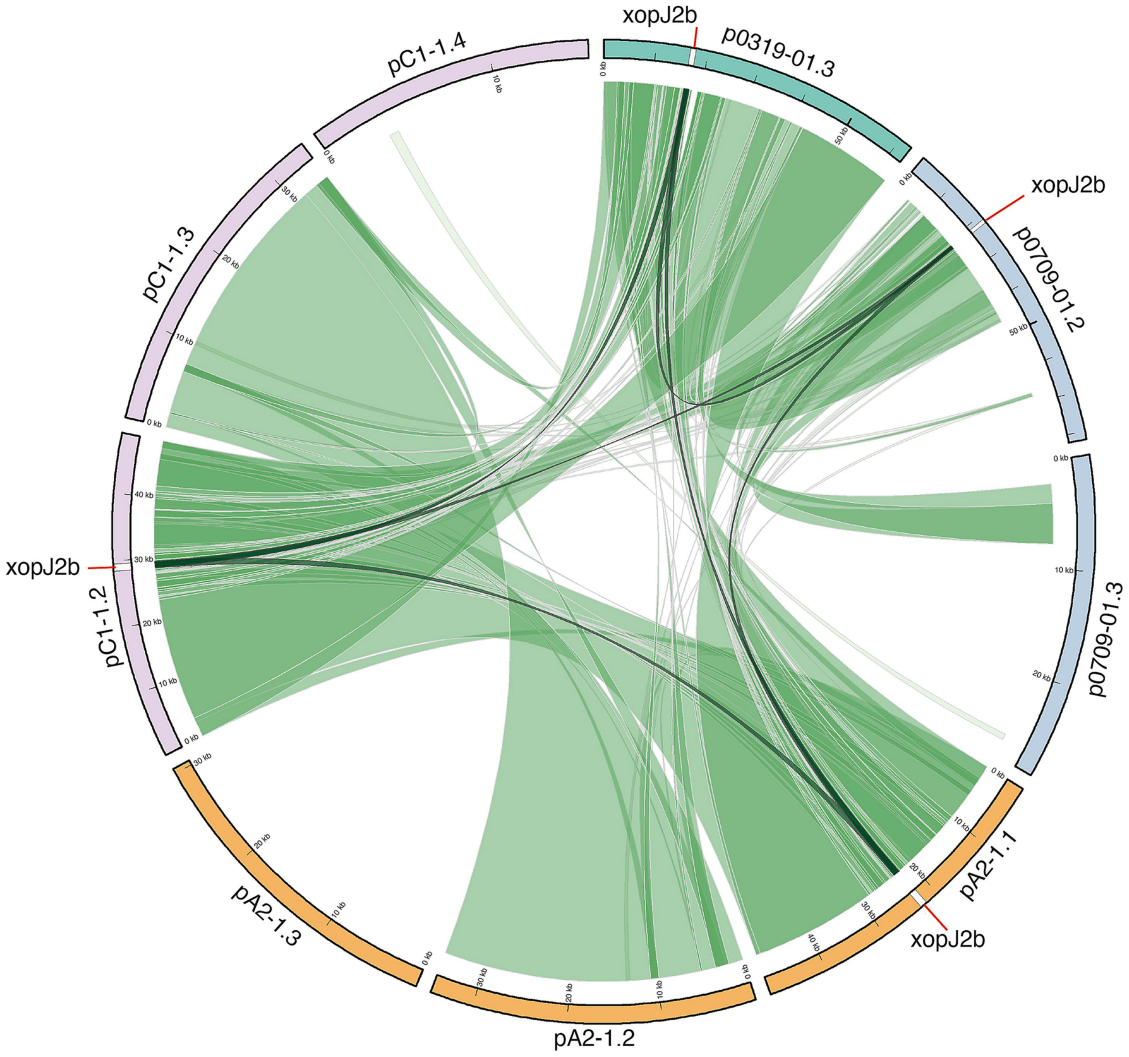
Comparative analysis of plasmid architecture and conservation of the *xopJ2b* locus. Plasmids from *Xep* race T2 strains A2-1 and C1-1 were compared with reference plasmids pT0319-01.3 (*Xet*) and p0709-01.3 (*Xep*) using nucmer (MUMmer v4). Pairwise homology is displayed with Circos: dark green ribbons represent regions with >90% nucleotide identity, and light green ribbons represent regions with 80–90% identity. The effector gene *xopJ2b* was consistently retained across plasmids, with its insertion sites highlighted by black connecting lines.

Despite the loss of *xopJ2a*, both genomes harbor its homolog *xopJ2b* (~71% amino acid identity), which is carried on a ~49-kb plasmid (Potnis et al., 2011; Iruegas-Bocardo et al., 2018; Jibrin et al., 2022). As previously observed in other *Xep* strains (Huang et al., 2024), the *xopJ2b* locus in A2-1 and C1-1 is flanked by insertion sequence elements (Figure 5 and Supplementary Figure S3), indicating that this gene was likely acquired through recombination. Taken together, the effector composition of A2-1 and C1-1 is consistent with recombination- and horizontal gene transfer-mediated turnover. The absence of *xopJ4*, the truncation of *xopAF*, and the plasmid-borne presence of *xopJ2b* illustrate the allelic variability of T3SEs within *Xep* tomato races.

### Plasmid diversity and evolution of heavy metal resistance gene clusters

Both A2-1 and C1-1 carried three completely circularized plasmids (Table 2). Two plasmids (49-kb and 34.6-kb) were shared by the two strains. Comparative analysis suggests that the 49-kb plasmid appeared to be a fused derivative from larger plasmids previously reported in Taiwanese strains, a 63-kb plasmid (pT0319-01.3) from *Xet* and an 82-kb plasmid (p0709-01.2) from *Xep* (Huang et al., 2024), likely through recombination and deletion (Figure 5). On the other hand, the 34.6-kb plasmid showed similarity to plasmid p40 of *Xep* WHRI 8957 (GenBank: CP167824; 100% coverage and 99.9% identity). In addition to these common plasmids, strain A2-1 carried a 30.4-kb plasmid closely related to p38 of *X. axonopodis* pv. *ricni* NCPPB 2369 (GenBank: CP167203; 92% coverage and 92.6% identity) and pE of *X. citri* pv. *phaseoli* var. *fuscans* CFBP6166 (GenBank: CP021004; 91% coverage and 91.8% identity) (Ruh et al., 2017). A unique 15.2-kb plasmid with no known homology in other strains was identified in C1-1.

Both A2-1 and C1-1 exhibited a copper resistant phenotype, but unlike previously reported *Xep* strains (Huang et al., 2024), neither genome carried the typical Cu^R^ megaplasmid (Table 2). Instead, an ~85 kb region of the chromosome harbored a complete set of heavy metal resistance gene cluster, corresponding to the previously identified Cu^R^ megaplasmid of *Xep* strain T0709-01 (Huang et al., 2024). This chromosomal cluster included genes for copper and mercury resistance as well as three ATP-binding cassette (ABC) heavy metal transporters (Huang et al., 2024). Consistent with this integration, no plasmid related to the canonical Cu^R^ plasmid was detected in either genome.

To assess the mobility of these resistance determinants, conjugation assays were performed between Cu^R^ donor strain A2-1 and Cu^S^ recipient XTN47Rif. No Cu^R^ transconjugants were detected, and the conjugation frequency was less than 10^−8^ transconjugants per recipient. Furthermore, strain A2-1 showed reduced growth on copper-amended medium compared with Cu^R^ strain T0709-01, which retains the Cu^R^ cluster on a megaplasmid. These findings suggest that the integration of heavy metal resistance clusters into the chromosome provides stable inheritance but may limit horizontal transfer and reduce the level of copper tolerance relative to a megaplasmid-borne resistance.

### Prophages and evolution of *Xep* race T2 strains

Our previous work showed that the *xopJ4* gene in *Xep* strains is consistently embedded between the *gpJ* and *gpS* genes within a P2-like temperate prophage region (Huang et al., 2024) (Figure 4A). In contrast, both A2-1 and C1-1 completely lacked the *xopJ4*-associated prophage, indicating the loss of the *xopJ4*-bearing element. Instead, another P2-like prophage, namely prophage XepTw1, was identified in the same chromosomal location. XepTw1 showed no significant sequence identity to the *xopJ4*-containing prophage and carried no predicted effector genes. A highly similar element to XepTw1 was also detected in *Xep* strain JK22-3 (GenBank: CP182560; 92% coverage and 97.69% identity).

**Figure 6:**
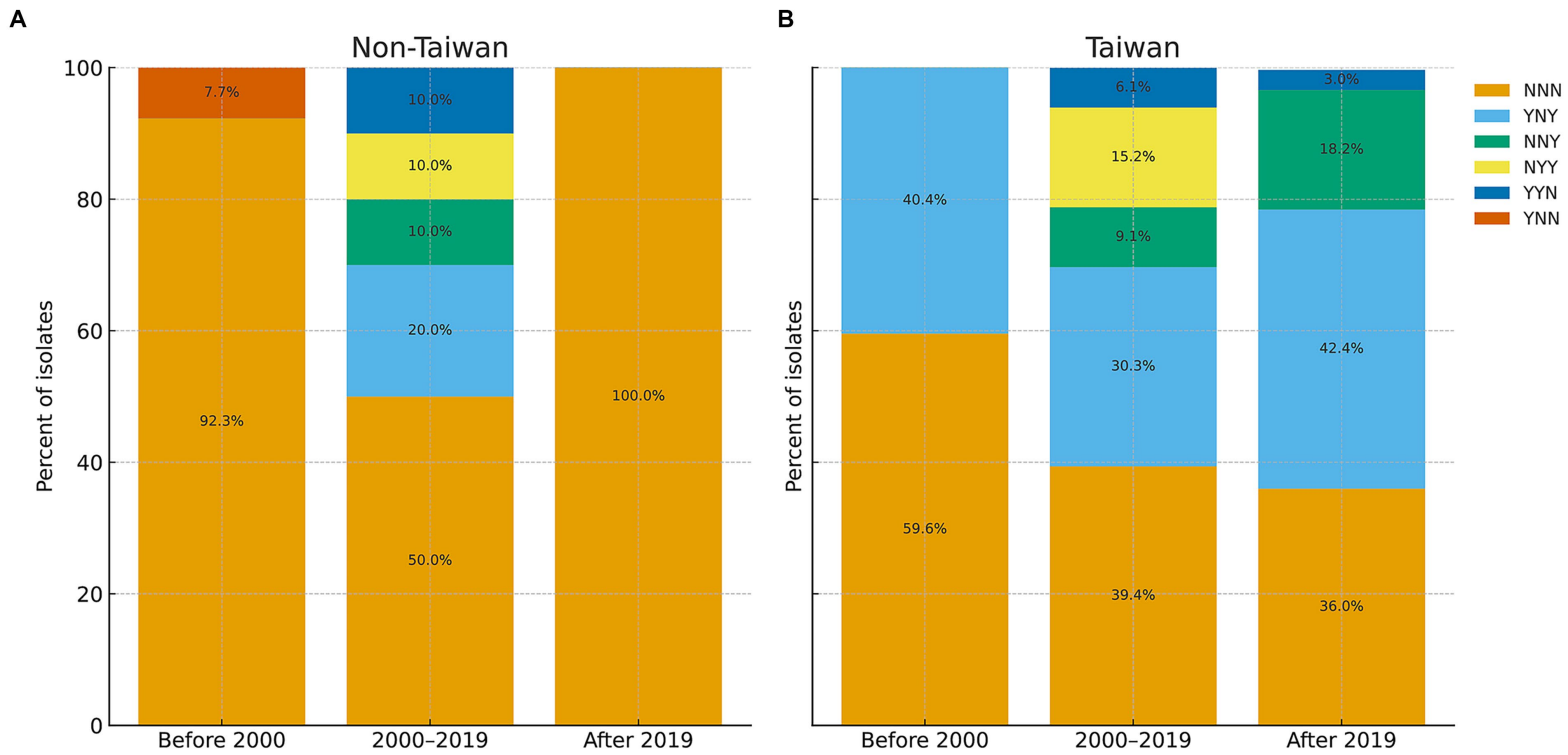
Temporal and geographic distribution of prophage profiles (XepTw2, XepTw3, XepTw4) in *Xep*. A total of 145 strains collected from **(A)** non-Taiwan and **(B)** Taiwan were screened for the presence or absence of prophage sequences XepTw2, XepTw3, and XepTw4. Stacked bar charts show the relative frequency of prophage profiles defined by the presence (Y) or absence (N) of XepTw2, XepTw3, and XepTw4. Each bar represents a collection period (before 2000, 2000–2019, after 2019), with separate panels for non-Taiwan and Taiwan isolates. Bar segments correspond to specific prophage profiles (e.g., NNN = absence of all three prophages, YNN = presence of XepTw2 only), and the height of each segment indicates the percentage of isolates carrying that profile. Labels are shown for segments representing of the population.

In addition to XepTw1, two complete prophages (XepTw2 and XepTw3) and one prophage remnant (XepTw4) were present in A2-1 and C1-1, flanking the ~85-kb chromosomal heavy metal resistance island near the replication terminus (Figure 4B). BLASTN comparison showed that XepTw2 was nearly identical to a prophage in *Xep* WHRI 8957 (GenBank CP167822; 100% coverage, 99.99% identity). In contrast, XepTw3 showed similarity to a prophage of *X. euvesicatoria* pv. *alfalfae* CFBP 3836 from Sudan (GenBank CP072268; 84% coverage, 90.60% identity), and XepTw3 was not detected in other tomato or pepper spot xanthomonads. Notably, A2-1 and C1-1 are the first *Xep* strains observed to harbor both XepTw2 and XepTw3. Moreover, intact XepTw2 elements were only identified in draft genomes of strains previously collected between 2005 and 2018, while XepTw3 had been seen only as fragmented remnants in *Xep* and *Xet* genomes (2005–2020) (Figure 6).

The results indicate that non-Taiwan isolates were consistently dominated by prophage-negative strains (NNN), while Taiwanese isolates displayed greater diversity, including combinations carrying XepTw3 or XepTw4 that increased in frequency after 2000. This highlights geographic and temporal differences in prophage turnover within *Xep* populations. In summary, comparative genomics indicates absence of the *xopJ4*-associated prophage and the presence of distinct P2-like elements (XepTw1–3) in these race T2 strains, consistent with prophage turnover at the *xopJ4* locus.

## Discussion

Tomato races of bacterial spot xanthomonads can be differentiated by inoculating a set of four tomato cultivars carrying defined resistance genes and observing either disease or a hypersensitive response (HR) (Jibrin et al., 2022). The race-associated effectors *avrRxv*, *xopAF* (syn. *avrXv3*), and *xopJ4* (syn. *avrXv4*) and their corresponding tomato resistance genes have been well characterized (Whalen et al., 1993; Astua-Monge et al., 2000a; Astua-Monge et al., 2000b; Stall et al., 2009). PCR-based assays targeting these loci enable rapid molecular profiling of these race-associated effectors (Bouzar, 1994; Timilsina et al., 2016; Klein-Gordon et al., 2021). In the view of races and bacterial spot xanthomonads, all race T1 and T2 strains were constantly identified as *X. euvesicatoria* pv. *euvesicatoria* (*Xee*) and *X. vesicatoria* (Osdaghi et al., 2021). In contrast, *Xep* populations have been highly diverse and previously assigned into races T3 and T4 (Osdaghi et al., 2021; Jibrin et al., 2022; Chen et al., 2024). Using differential cultivar phenotyping, we defined these two Taiwanese isolates of *X. euvesicatoria* pv. *perforans* (*Xep*) as race T2; effector profiles and complete genomes provided supporting evidence consistent with this phenotype. No HR formed on differential cultivars reflected the strains with race T2 phenotype, and both genomes lacked *avrRxv* and *xopJ4* and carried a truncated *xopAF*, corroborating this assignment. Whole-genome ANI/dDDH analyses further confirmed that these strains belong to the *Xep* lineage (Figure 1 and Supplementary Figure S1). This finding extends the known race spectrum of *Xep* to include T2.

Routine surveillance integrating PCR-based effector genotyping with whole-genome analyses will be essential for tracking effector shifts in field populations. Such monitoring can help anticipate breakdowns of host resistance and guide breeding strategies for durable resistance. Because no effective resistance gene is currently available against race T2 (Stall et al., 2009; Jibrin et al., 2022), the emergence of *Xep* T2 represents an increasing threat to tomato production. Our DeepSecE analysis further suggests that *Xep* may harbor a latent pool of candidate effectors, including lineage-specific genes annotated as hypothetical proteins with strong effector signals; these candidates may represent accessory virulence factors acquired via horizontal gene transfer that contribute to host adaptation or immune evasion rather than defining race phenotypes. These predictions are hypothesis-generating and require functional validation (e.g., targeted mutagenesis and host-range assays). Collectively, this argues that PCR assays targeting only a small set of known effectors are unlikely to be sufficient for monitoring emerging threats. Beyond surveillance, future work should prioritize (i) functional validation of putative effectors and copper resistance (Cu^R^) mechanisms (e.g., targeted mutagenesis) and (ii) time-resolved (longitudinal) genomic sampling to test directionality and confirm inferred evolutionary trajectories.

### Plasmids as a stable vehicle for virulence factors

Plasmids in tomato- and pepper-pathogenic xanthomonads are highly diverse in size and often carry key virulence and avirulence genes (Minsavage et al., 1990; Thieme et al., 2005; Roach et al., 2019; Huang et al., 2021; Huang et al., 2024; Kaur et al., 2025). A prior study reported a 63-kb plasmid in a Taiwanese *Xet* (atypical *X. euvesicatoria*) strain and an 82-kb plasmid in a *Xep* strain that likely merged via recombination in a co-integrate plasmid (Huang et al., 2024). This phenomenon of plasmid fusion was also observed in *X. citri* strains from Taiwan (Huang et al., 2021), indicating that plasmid-plasmid recombination is a recurring theme in *Xanthomonas* evolution. In this study, we identified a ~49-kb plasmid that appears to be a recombinant derivative of the aforementioned 63-kb and 82-kb plasmids, with some regions lost during the fusion/deletion process (Figure 5). Despite the reduction in size, this 49-kb plasmid retains the intact *xopJ2b* effector gene. Strikingly, *xopJ2b* was present on all plasmid size variants (82, 63, and 49 kb) and was always flanked by two insertion sequences, as previously noted. The preservation of *xopJ2b* through these plasmid rearrangements underscores the importance of this effector to the pathogen. We infer that plasmids serve as stable vehicles for transmitting virulence factors like *xopJ2b* among xanthomonads, even as they undergo recombination and size reduction. Maintaining a smaller plasmid might even confer a fitness advantage (lower replication cost) while still carrying essential effectors (San Millan and MacLean, 2017). Overall, the diverse virulence plasmids in *Xep* and *Xet* act as mobile genetic platforms that can disseminate effector genes across strains, thereby shaping the pathogenic potential and host range (Abrahamian et al., 2018; Huang et al., 2024). Plasmid-mediated horizontal gene transfer has likely contributed to the rapid shifts in pathogen genotype observed in the field (Abrahamian et al., 2018; Huang et al., 2024; Kaur et al., 2025), emphasizing the need to consider plasmid profile when tracking pathogen evolution (Timilsina et al., 2025).

### Evolution and loss of type III effectors via mutation and lysogenic conversion

Multiple mechanisms drive the gain or loss of type III secretion effectors (T3SEs) in *Xanthomonas*, including point mutation, transposon insertion, recombination, and lysogenic conversion by bacteriophages (Timilsina et al., 2016; Huang et al., 2021; Chen et al., 2024; Huang et al., 2024). Our analysis provides new examples of these processes. In the *Xep* race T2 strains, the *xopAF* gene (*avrXv3* effector) contained an early stop codon mutation (Astua-Monge et al., 2000a). This nonsense mutation would truncate XopAF and render it nonfunctional, which is consistent with the strain’s ability to evade the tomato *Xv3* resistance gene (Timilsina et al., 2016; Chen et al., 2024). Similar *xopAF* disruptions were previously reported in race T4 strains from Florida, where *avrXv3* was inactivated by frameshifts or insertion sequence (IS) element insertions (Timilsina et al., 2016). By losing a functioning *avrXv3*, *Xep* strains gain the ability to infect *Xv3*-resistant tomatoes because they no longer trigger the host defense response (Timilsina et al., 2016). This illustrates how agricultural use of resistance genes selects for pathogen mutants with effector loss-of-function mutations (Thrall et al., 2011). In contrast, new effectors can be gained via horizontal gene transfer, as seen with *xopJ4* carried on a P2-like prophage in race T3 and T4 strains (Huang et al., 2024). Our comparative genomics now shows that two race T2 strains lack the *xopJ4*-carrying prophage. Instead, a different prophage occupies the same chromosomal locus without effector genes. This replacement indicates that lysogenic conversion facilitated not only the acquisition of *xopJ4* in other *Xep* lineages but also its loss, likely favoring strains’ ability to evade recognition by *Xv4*-containing tomato cultivars (Askora et al., 2017; Harrison and Brockhurst, 2017; Greenrod et al., 2022).

In addition to these prophage-mediated changes, our analysis of virulence plasmids revealed another layer of effector mobility. The *xopJ2b* gene in race T2 strains was consistently located on the 49-kb plasmid, always flanked by IS elements, as reported previously (Huang et al., 2024). IS-mediated transposition has been implicated in the gain and loss of *xopJ2a* (*avrBsT*) and *xopJ2b* across *Xep* and *Xet* populations (Klein-Gordon et al., 2021; Huang et al., 2024). Both effectors can trigger hypersensitive responses in *Arabidopsis* and pepper (Minsavage et al., 1990; Timilsina et al., 2016; Abrahamian et al., 2018; Iruegas-Bocardo et al., 2018; Sharma et al., 2024), restricting host range primarily to tomato. The consistent retention of *xopJ2b* on diverse plasmids, despite recombination and deletion events, suggests that this effector provides an important fitness benefit to *Xep* and *Xet* during interaction with host plants.

### Evolution of copper resistance in *X. euvesicatoria* pv. *perforans* in Taiwan

Over decades, the heavy use of copper-based bactericides to control bacterial spot has driven the emergence of copper-resistant (Cu^R^) strains of *Xanthomonas* in many regions (Kaur et al., 2025). Our previous work showed that Cu^R^ strains of *Xep* and *Xet* in Taiwan harbored a ~ 250 kb conjugative megaplasmid carrying the *copLAB* operon and associated resistance genes (Huang et al., 2021). This plasmid conferred high copper tolerance and enabled survival under bactericide sprays. However, in the current study the two Cu^R^ Xep strains we sequenced lacked this megaplasmid. Instead, they prossessed an ~85 kb genomic island integrated into the chromosome, containing a complete cluster of heavy metal resistance genes (*copLABGF* and additional determinants). This finding suggests an evolutionary trajectory in which plasmid-borne resistance islands became fixed on the chromosome, possibly as a trade-off between resistance level and fitness cost. Both plasmid-borne and chromosomal Cu^R^ loci provide an advantage under copper stress, but their levels of effectiveness differ. Recent surveys of *X. perforans* in Florida found that chromosomal Cu^R^ loci predominate, yet strains retaining plasmid-borne clusters showed higher copper tolerance *in vitro* (Kaur et al., 2025), consistent with our observation that the Taiwanese strains with only chromosomal Cu^R^ islands exhibited slightly lower tolerance.

These results highlight how agricultural practices shape pathogen evolution whereas under intense copper usage, plasmid-borne resistance provides strong protection, but when fitness costs are high or copper use is reduced, chromosomal integration may be favored (Behlau et al., 2013; Marin et al., 2019; Kaur et al., 2024). Monitoring whether resistance genes are plasmid- or chromosome-encoded will be critical for disease management, as plasmid-borne loci remain highly mobile, while chromosomal integration indicates more stable, lineage-specific adaptation (Potnis, 2021; Chen et al., 2024). Collectively, these findings reshape our understanding of *Xanthomonas* evolution by demonstrating that effector repertoire plasticity and copper resistance can rapidly transition from unstable mobile determinants to fixed chromosomal and prophage-driven architectures. This transition significantly contributes to the pronounced diversity within *Xep* race populations and substantially reduces the long-term efficacy of copper-base bactericides and single-gene host resistance. This underscores the critical need for integrated management strategies that avoid reliance on any single control measure for bacterial spot and similar *Xanthomonas*-associated diseases. Taken together, the effector mutations (Klein-Gordon et al., 2021; Timilsina et al., 2025), prophage-mediated turnover (Greenrod et al., 2022; Rana et al., 2022), plasmid rearrangements (Huang et al., 2021; Huang et al., 2024), and chromosomal integration of resistance clusters all point to a common theme: recombination and horizontal gene transfer are the major forces shaping the evolution of *Xep* race T2, enabling rapid adaptation to host resistance and disease management strategies.

## Conclusion

Using complete, gap-free genome assemblies and differential-cultivar assays, we verified that two Taiwanese *Xanthomonas euvesicatoria* pv. *perforans* (*Xep*) isolates are tomato race T2. Effector mining provided the diagnostic profile, absence of *avrRxv* and *xopJ4* with a truncated *xopAF*, and phylogenomics analysis placed both strains within *Xep*. Comparative analyses against published *Xep* genomes revealed extensive plasmid restructuring and clear signatures of recombination and horizontal gene transfer, including prophage replacement at the *xopJ4* locus. The virulence effector *xopJ2b* was consistently maintained on a ~ 49-kb plasmid and flanked by insertion sequences, indicating a mobile but conserved fitness determinant. Both strains lacked a Cu^R^ megaplasmid. Instead, an ~85-kb chromosomal island carrying complete copper and mercury resistance clusters and multiple ABC transporters was integrated into the chromosome. This configuration points to ongoing remodeling of heavy-metal resistance, with chromosomal fixation providing stability while potentially reducing horizontal mobility relative to plasmid-borne loci. Taken together, the data indicate that lysogenic conversion, IS-mediated transposition, and plasmid recombination jointly shape the genome architecture of emerging *Xep* race T2 lineages. In the absence of an effective tomato resistance gene against race T2, sustained field surveillance that couples effector genotyping with genome-resolved tracking of resistance loci (plasmid versus chromosome) is warranted to anticipate adaptation and to inform breeding and management in tomato production systems.

## Data Availability

The datasets presented in this study can be found in online repositories. The names of the repository/repositories and accession number(s) can be found at: NCBI BioProject PRJNA1313807 https://github.com/YaoChengLab/MicroGenomePipeline/ https://doi.org/10.5281/zenodo.17900872.

## Footnotes

1
https://github.com/YaoChengLab/MicroGenomePipeline/

2
https://github.com/rambaut/figtree/releases
